# How Reproducibility Will Accelerate Discovery Through Collaboration in Physio-Logging

**DOI:** 10.3389/fphys.2022.917976

**Published:** 2022-07-08

**Authors:** Max F. Czapanskiy, Roxanne S. Beltran

**Affiliations:** ^1^ Hopkins Marine Station, Stanford University, Pacific Grove, CA, United States; ^2^ Department of Ecology and Evolutionary Biology, University of California Santa Cruz, Santa Cruz, CA, United States

**Keywords:** bio-logging, ecophysiology, ecoinformatics, cyberinfrastructure, technical debt

## Abstract

What new questions could ecophysiologists answer if physio-logging research was fully reproducible? We argue that *technical debt* (computational hurdles resulting from prioritizing short-term goals over long-term sustainability) stemming from insufficient *cyberinfrastructure* (field-wide tools, standards, and norms for analyzing and sharing data) trapped physio-logging in a scientific silo. This debt stifles comparative biological analyses and impedes interdisciplinary research. Although physio-loggers (e.g., heart rate monitors and accelerometers) opened new avenues of research, the explosion of complex datasets exceeded ecophysiology’s informatics capacity. Like many other scientific fields facing a deluge of complex data, ecophysiologists now struggle to share their data and tools. Adapting to this new era requires a change in mindset, from “data as a noun” (e.g., traits, counts) to “data as a sentence”, where measurements (nouns) are associate with transformations (verbs), parameters (adverbs), and metadata (adjectives). Computational reproducibility provides a framework for capturing the entire sentence. Though usually framed in terms of scientific integrity, reproducibility offers immediate benefits by promoting collaboration between individuals, groups, and entire fields. Rather than a tax on our productivity that benefits some nebulous greater good, reproducibility can accelerate the pace of discovery by removing obstacles and inviting a greater diversity of perspectives to advance science and society. In this article, we 1) describe the computational challenges facing physio-logging scientists and connect them to the concepts of *technical debt* and *cyberinfrastructure*, 2) demonstrate how other scientific fields overcame similar challenges by embracing computational reproducibility, and 3) present a framework to promote computational reproducibility in physio-logging, and bio-logging more generally.

## Introduction

Ecophysiology, like many other scientific disciplines, is undergoing a technological revolution fueled by advances in both hardware and software. One technology dramatically advancing the research boundary is physio-logging, which enabled observations of animals’ physiology in the field using animal-borne sensors (Fahlman et al., 2021; Hawkes et al., 2021). Ecophysiologists suddenly find themselves with far more data, of increasing complexity, than ever before. Although these new data led to breakthroughs in answering individual questions, the lack of field-wide standards and tools largely siloed research groups, stifling collaboration and synthesis.

In this article, we introduce the concepts of *cyberinfrastructure* and *technical debt* (Table 1) as they apply to physio-logging research using a trio of researcher personas and the scientific challenges they face. Then, we describe how other scientific disciplines, such as astronomy, neuroscience, and molecular biology, have addressed these issues by embracing reproducibility as a guiding principle. Finally, we propose physio-logging cyberinfrastructure that promotes reproducibility and reduces barriers to collaboration.

**TABLE 1:** Glossary of terms.

Term	Definition	References
Cyberinfrastructure	The collective interface between data collection and data analysis for a scientific field, including software, hardware, personnel, and shared practices	Atkins et al. (2003)
Technical debt	Short-term, sub-optimal choices in data and code that hamper future development without refactoring, such as missing documentation and bug-prone code	(Hinsen, 2015; Codabux et al., 2021; Vidoni, 2021)
Heterogeneous data	Combinations of data collected at different temporal scales and/or with different properties, for example multivariate time series (e.g., acceleration) with intermittent geospatial locations (e.g., GPS)	(Leinfelder et al., 2011; Michener and Jones, 2012)
Literate programming	A programming technique that combines code itself with descriptive text and outputs (figures, tables). R Markdown is an implementation of literate programming	(Knuth, 1984; Baumer and Udwin, 2015; Kery et al., 2018)
Data provenance	A record of the origin and processing steps that produced the data	Michener and Jones, (2012)

### Barriers to Collaboration and Synthesis

In the following vignettes, three physio-logging researcher personas encounter common scientific challenges stemming from accumulated technical debt and insufficient cyberinfrastructure. We expect these challenges will be familiar to the reader and help connect their own experiences to the concepts in this article.

*Person A is a graduate student studying ecophysiology. They have their own physio-logging data and a second dataset contributed by a collaborating lab. Before conducting any meaningful analysis, Person A spends multiple weeks corresponding with another grad student in the collaborating lab to figure out how to merge the datasets due to mismatched variable names and units. Later in the analysis, Person A discovers that their own data recorded time in Coordinated universal Time and the collaborating lab used local time, requiring them to spend more time correcting the input data and re-running earlier steps. The analysis is not ready in time for their next committee meeting.*

*Person B is the Principal Investigator of a comparative physiology lab. The lab recently completed a field season and the trainees are working hard to process all the new physio-logging data. Meanwhile, Person B wants to know how their study system fits into the broader phylogenetic and morphological space, so they ask comparable physio-logging data from other labs. Despite a rich literature on this subject, only a small number of labs have the bandwidth and/or interest to contribute data for a synthesis study. As a result, Person B writes a paper with limited comparative power and they struggle to find funding for the next field season.*

*Person C is a data scientist at a government agency developing a new deep learning method for time series classification. They meet Person A at a café on campus and realize their physio-logging data would be a perfect case study for the new method. Unfortunately, the dataset is not large enough on its own, so Person A promises to contact their collaborators to get more data. The data management issues encountered earlier by Person A present such substantial barriers to collating a sufficiently large dataset that both Persons lose interest and move on to other projects. Person C’s method contributes to breakthrough advances in bio-medical research, but they never think about physio-logging again.*

The challenges encountered by the researcher personas illustrate how technical debt prevents collaboration and innovation. *Person A* is the early career researcher who works hands-on with bio-logging data, navigating a fragile computational ecosystem of inconsistently formatted data and poorly documented scripts. *Person B* is further along in their career and delegates computational tasks to achieve their scientific goals. *Person C* represents scientists in other fields who could make important contributions to physio-logging if they were given access and resources. The obstacles facing each persona are unique to their perspective and experience, but they all have the same consequence: narrowing the scope of scientific inquiry.

### Cyberinfrastructure and Technical Debt

*Cyberinfrastructure* is a layer in the technological stack driving modern science, situated between data production and discipline-specific analysis practices (Atkins et al., 2003). Translated into the physio-logging domain, “data production” includes electronic tags deployed on animals in the field. “Analysis practices” refers to both technical (e.g., statistical methods and packages) and social (e.g., norms about sharing data and code) aspects of physio-logging science. Cyberinfrastructure is the oft invisible middle layer that interfaces between data production and analysis practices. Efforts towards cyberinfrastructure in bio-logging generally include, for example, universal data standards (Campbell et al., 2016; Sequeira et al., 2021) and repositories with interfacing software (Kranstauber et al., 2011; Kays et al., 2022). The purpose of cyberinfrastructure is to “provide an effective and efficient platform for the empowerment of specific communities of researchers to innovate and eventually revolutionize what they do, how they do it, and who participates” (Atkins et al., 2003, p. 5).

In the absence of cyberinfrastructure, scientists are incentivized by funding, hiring, and promotion structures to choose rapid data collection and analysis over long-term technical sustainability. *Technical debt* is the lingering bug, the missing documentation, the “I swear it works on my machine” left behind in pursuit of scientific output (Hinsen, 2015). Up to a point, technical debt is not a problem; rather, it is the natural side effect of important scientific tasks like exploratory analyses and prototyping tools. But eventually technical debt creates obstacles for future work (Codabux et al., 2021; Vidoni, 2021). Removing those obstacles requires either 1) resources allocated specifically to auditing and fixing data and code (i.e., refactoring Adorf et al. (2019)) or 2) cyberinfrastructure that promotes best practices from the start. The first approach is unlikely to work at scale because existing incentive structures (funding, publications, hiring and promotion) do not prioritize refactoring existing data and code. Other scientific fields embraced the second approach, improved cyberinfrastructure, to address the same issues facing physio-logging. But before we investigate those efforts, we first describe in greater detail the technical debts in physio-logging.

### Where Did Our Technical Debt Come From

The underlying cause of the obstacles to collaboration and synthesis is a field-wide, collective technical debt incurred when an empirical/theoretical science (ecophysiology) embraced a data-intensive method (physio-logging) without simultaneously developing cyberinfrastructure. What was it about physio-logging that triggered ecophysiology’s technical debt?

Physio-logging changed the nature of ecophysiological data from simple, small tables to dense, *heterogeneous* (Table 1) mixtures of physiological, geospatial, and biomechanical time series (Harrison, 2021). Major developments in ecophysiology during the 20th and early 21st centuries were fueled by comparative analyses across morphology, phylogeny, geography, and other biological dimensions. Critically, these comparative studies were only possible because data could be combined from multiple individual studies. Consider the Metabolic Theory of Ecology, which emerged from scaling approaches that connected patterns and processes across the many levels of biological organization, from molecules to ecosystems (Brown et al., 2004). Three *Persons A* (Gilooly, Allen, and Savage–post-doctoral scholars at the time) and a *Person B* (Brown–an established biologist) assembled a synthetic dataset from dozens of earlier publications. In collaboration with a *Person C* (West–a theoretical physicist), the group articulated a theoretical framework for ecology from first principles. Though controversial, the Metabolic Theory of Ecology inspired a wave of innovative research (Lafferty et al., 2008; Burton et al., 2011; Gardner et al., 2011; Locey and Lennon, 2016) and amounted to a major advance in biological theory. Coincidentally, while Brown et al. were analyzing “simple” ecophysiological data, physio-logging data were growing in size and complexity. No longer simple recorders that measured individual data points (Kooyman, 1966; Goldbogen and Meir, 2014), by 2004 bio-loggers had evolved into high-resolution, multisensory devices collecting gigabytes of heterogeneous data (Wilmers et al., 2015). A scientific community accustomed to simple, small datasets was suddenly presented with a profound informatics challenge–largely without the training, resources, and incentives to properly meet it. We have yet to develop adequate cyberinfrastructure, leaving a growing technical debt that impedes *Persons A-C*’s progress and limits discoveries using physio-logging tools.

### Reproducible Research Repays Technical Debt

As biologists, we are trained to think of data as observations. In other words, data are nouns: a cow’s mass, a salmon’s heart rate, a bird’s body temperature. However, physio-loggers record such vast quantities of complex, heterogeneous data that their interpretation *cannot be separated* from the computational methods used to process and analyze them. For example, consider a table of 50 Hz tri-axial accelerometer and magnetometer data. Even when visualized graphically, these data are largely meaningless to a human interpreter. But if we correct for the orientation of the tag relative to the animal’s body and account for the declination and inclination of the local magnetic field, then we can transform acceleration and magnetism into pitch, roll, and heading (Johnson and Tyack, 2003). Now the human interpreter can meaningfully visualize the animal’s fine-scale movements to identify physiologically relevant behaviors such as exercise (Williams et al., 2020), rest (Mitani et al., 2010), and escape (Williams et al., 2017). Physio-logging “data” are more than the final numbers; they are also the computational pipeline that transforms raw measurements into useful information. When it comes to physio-logging, data are not nouns; they are whole sentences composed of verbs (transformations), adjectives (metadata), and adverbs (parameters).

Computational reproducibility is the practice of writing the data’s entire sentence so that anyone can read it. Sharing our data this way accomplishes both “global” and “local” goals (Feinberg et al., 2020). The “global” goal is satisfying the scientific norm of reproducibility, providing transparency and integrity for our entire field. These ideals are the focus of the widely reported “reproducibility crisis” (Peng, 2015; Fanelli, 2018). But other data-intensive fields have embraced a “local” goal for computational reproducibility by reframing it in terms of collaboration and knowledge transfer. In this context, reproducibility is accomplished through computational best practices, such as re-usable code and proper documentation. This framing contains tangible solutions to the challenges experienced by *Persons A-C*. Sharing data as reproducible workflows (Cohen-Boulakia et al., 2017; Grüning et al., 2018; Wratten et al., 2021) solves the incompatibility issues that distracted *Person A* from doing good science and promotes collaboration within (*Person B*) and between (*Person C*) scientific disciplines. More importantly, it removes technical obstacles preventing broad, equitable access to our science. But a “data as a sentence”, reproducibility-focused mindset is too much to expect of individual ecophysiologists without adequate cyberinfrastructure to support them. How have other fields provided tools, education, and other resources to their scientists?

### Cyberinfrastructure Examples From Other Fields

Most scientific fields are facing similar informatics challenges as ecophysiology; a few have developed cyberinfrastructure to promote sharing “data as a sentence”. What physio-logging is to ecophysiology, sky imaging is to astronomy, brain imaging is to neuroscience, and high-throughput sequencing is to molecular biology. The quantity and complexity of image and sequence data created technical debts in those fields as well, which were addressed through the coordinated development of cyberinfrastructure. Saliently, these fields used the “local” goal of computational reproducibility (collaboration and knowledge transfer) to motivate adoption of their respective cyberinfrastructures.

Astronomy’s cyberinfrastructure is represented by the aptly named Virtual Observatory (VO) (Quinn et al., 2004). There are currently 22 VOs around the world, such as the National Virtual Observatory in the United States and the Virtual Observatory of India. Each VO provides open access to sky imagery and other astronomical data in standardized formats agreed upon by the International Virtual Observatory Alliance (IVOA). In addition to data sharing, VOs provide open processing and analysis workflows, providing astronomers with invaluable tools for the “data as a sentence” mindset (Cui et al., 2020). The VO framework fuels innovative breakthroughs in astronomy, including the discovery of galaxies (Chilingarian et al., 2009).

In neuroscience, the proliferation of brain image data led to ad hoc, incompatible data curation practices, inspiring a standardization effort: the Brain Imaging Data Structure (BIDS). BIDS was developed to solve a problem familiar to physio-logging scientists: “Lack of consensus leads to misunderstanding and time wasted on rearranging data or rewriting scripts that expect particular file formats and organization, as well as a possible cause for errors.” (Gorgolewski et al., 2016, p. 2). In addition to a standardized data format, BIDS includes a software ecosystem for importing, validating, and processing imaging data (Gorgolewski et al., 2017). Like VOs, BIDS integrates data and code, facilitating “data as a sentence”.

The Human Genome Project published a draft of the human genome in 2001, representative of molecular biology’s pivot to big data (Lander et al., 2001). Three years later, Bioconductor emerged as a provider of data access, processing, and analysis tools (Gentleman et al., 2004; Huber et al., 2015). Like the VOs and BIDS, Bioconductor provides both data structures and computational methods. From the beginning, it was designed with reproducibility as an explicit goal, to promote collaboration in both sharing data and developing methods. As important new technologies emerge, such as high-throughput single-cell sequencing, the cyberinfrastructure provided by Bioconductor promotes collaborations between biologists, statisticians, and computer scientists to rapidly develop new methods for handling the influx of increasingly heterogeneous data (Amezquita et al., 2020). In turn, the cultural norm of reproducibility incentivizes best practices, such as software documentation and validation, that facilitate adoption by researchers across the discipline.

VOs, BIDS, and Bioconductor exemplify the “data as a sentence” perspective by providing both data formats and computational tools for handling large quantities of heterogeneous data. Although there are data formats (Sequeira et al., 2021; Kays et al., 2022) and computational tools (Joo et al., 2020) for geospatial bio-logging data, the two sides have been developed independently without shared interfaces. Physio-logging lacks any such infrastructure.

## Introducing biologr

What would physio-logging cyberinfrastructure look like in practice? Following the lessons of the Virtual Observatory, BIDS, Bioconductor, and other successful efforts, it should facilitate reproducibility by integrating data formats directly with computational tools. Beginning with the data format, the nc-eTAG specification provides an extensible and efficient structure for storing bio-logging data (Tsontos et al., 2020). But recall the data alone are only the nouns; we must also capture the rest of the sentence. Existing workflows for processing bio-logging data are typically ad hoc and ephemeral, meaning they’re lost from the scientific record after publication. As a result, the data shared in repositories, whether they’re specialized for bio-logging like MoveBank or general purpose like Zenodo, are missing much of the story.

**FIGURE 1:**
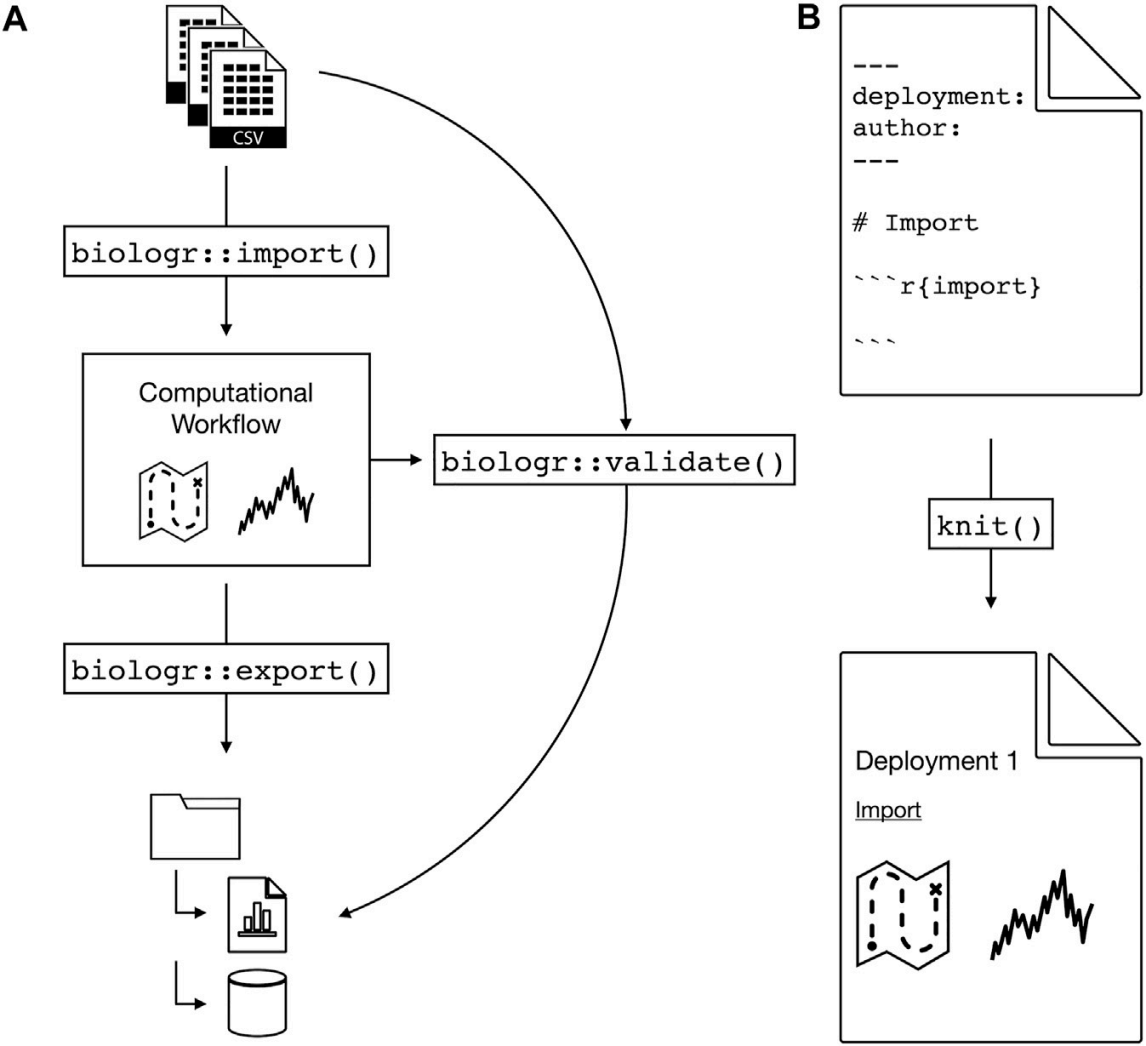
The proposed biologr R package will provide physio-logging cyberinfrastructure. **(A)** From raw data to standardized, reproducible data. Import and export functions ensure data standard compliance, e.g., file formats and directory structures. A validate function automatically verifies that the computational workflow (see **(B)**) reproducibly generates the processed data. **(B)** biologr provides an R Markdown template (see section Introducing biologr) for recording the workflow. The R Markdown knit command creates a report documenting data processing and interpretation, i.e., data provenance.

We propose an R package, biologr, that advances the goal of bio-logging cyberinfrastructure, supporting ecophysiologists and our colleagues in the broader bio-logging space. biologr interacts with the nc-eTAG data format and explicitly captures the entire workflow. By archiving both raw and processed data together with the workflow connecting them, biologr records the data’s entire sentence: nouns, verbs, and all (Figure 1).

What minimum set of functionalities would biologr require to make physio-logging data reproducible? In brief: data standards, import/export, validation, and workflow. For data standards, biologr relies on nc-eTAG for processed data and a standardized directory structure that organizes components for reproducibility. biologr needs both import and export functions to get the raw data into the nc-eTAG format and to populate the directory structure. Importantly, the export function saves both the data *and the computational workflow*. BIDS and other cyberinfrastructures have demonstrated that another critical function is automated validation (Gorgolewski et al., 2016). Computational reproducibility is built on standards, and without validation standardization quickly falls apart. So biologr’s automated validator checks both form (i.e., do the file formats and directory structures adhere to the standard?) and function (i.e., does the workflow reproducibly generate the processed data from the raw?). Import/export and validation both require a reproducible workflow, which is the component most responsible for reproducibility.

biologr captures the physio-logging computational workflow using *literate programming* (Table 1) (Knuth, 1984; Kery et al., 2018). Literate programming is a technique that interweaves code, text, and output (e.g., figures and tables) - a combination that captures computational workflows in both human- and computer-readable formats. biologr provides an R Markdown template, a literate programming implementation with broad adoption in the scientific community (Baumer and Udwin, 2015). Instead of an ad hoc, ephemeral script, the physio-logging researcher authors their workflow in R Markdown. Not only does this preserve computational details for automated validation and re-use/modification by other researchers, the output of the R Markdown file is itself a *provenance* report (Table 1), explicitly recording the transformations and interpretations in the data (Michener and Jones, 2012). This workflow approach promotes reproducibility by serving as the glue that binds together data standards, import/export, and validation.

## Discussion

### A Vision for the Future of Physio-Logging

The goal of this article is to invite the physio-logging community to a conversation about the existing limitations, and potential advances, in our work relating to cyberinfrastructure and reproducibility. The International Virtual Observatory Alliance, Brain Imaging Data Structure, and Bioconductor all have governing bodies with working groups to design, develop, and disseminate cyberinfrastructure for their fields. A working group within the International Bio-logging Society (www.bio-logging.net) could serve the same role for physio-logging, especially if ecoinformaticians were included in the process (Michener et al., 2012). The proposed biologr package provides a starting point for those conversations. Below, we offer alternative vignettes to describe how our researcher personas would benefit from biologr.

*Person A is a* *grad* *student studying ecophysiology. They have their own physio-logging data and a second dataset contributed by a collaborating lab. The two labs use slightly different methods to process their data, but both use* biologr *for recording their workflows. Person A edits their collaborator’s workflow to make the two datasets compatible and kicks off the reproducible workflow with a single click. They leave for lunch and come back to the lab to find two happily interoperable datasets. The analysis is done by the end of the week and their committee congratulates them on their progress.*

*Person B is the Principal Investigator of a comparative physiology lab. The lab recently completed a field season and the trainees are working hard to process all the new physio-logging data. Meanwhile, Person B wants to know how their study system fits into the broader phylogenetic and morphological picture. There is a rich literature on this subject, so dozens of archived datasets are available through a physio-logging data portal based on* biologr *. Person B downloads the data, runs a phylogenetically-informed scaling analysis, and writes a high-impact synthesis study. The publication opens exciting lines of collaborative inquiry and leads to a new multi-million dollar grant.*

*Person C is a post-doc in computer science developing a new deep learning method for time series classification. They meet Person A at a cafe on campus and realize their physio-logging data would be a perfect case study for the new method. Person C spends the afternoon reading* biologr *documentation and assembles a large physio-logging dataset from multiple studies. They demonstrate that their method accurately identifies physiological states from behavioral data and publish the method as a* biologr *-compatible R package, which is used by several physio-logging labs in their research.*

## Conclusion

Physio-logging, and bio-logging more generally, gave biologists new tools for observing animals in their natural habitats with previously inconceivable detail. But concurrent with this great leap forward, we accumulated a technical debt that cost us the ability to easily share and synthesize our data. Ecophysiology became a data-intensive science without developing the cyberinfrastructure to handle large quantities of complex, heterogeneous data. biologr illustrates how we can develop tools to reproducibly process and archive physio-logging data. By embracing reproducibility, we can repay our technical debt and usher in a new era of collaboration while fostering best practices in a diverse next generation of physio-logging researchers. As individuals, labs, data repositories, and other stakeholders adopt shared standards, the possibilities for exploration and synthesis will grow exponentially.

## Data Availability

The original contributions presented in the study are included in the article/Supplementary Material, further inquiries can be directed to the corresponding author.
